# Comparison of haplotyping methods using families and unrelated individuals on simulated rheumatoid arthritis data

**DOI:** 10.1186/1753-6561-1-s1-s55

**Published:** 2007-12-18

**Authors:** Xin Li, Jing Li

**Affiliations:** 1 Department of Electrical Engineering and Computer Science, Case Western Reserve University, 10900 Euclid Avenue, Cleveland, Ohio 44106, USA

## Abstract

In this report, we compared haplotyping approaches using families and unrelated individuals on the simulated rheumatoid arthritis (RA) data in Problem 3 from Genetic Analysis Workshop (GAW) 15. To investigate these two approaches, we picked two representative programs: PedPhase and fastPHASE, respectively, for each approach. PedPhase is a rule-based method focusing on the haplotyping constraints within each pedigree and solving them using integer linear programming. fastPHASE is a statistical method based on the clustering property of haplotypes in a population over short regions. It is believed that with family information, one can obtain more accurate phasing results with considerably more cost for genotyping additional family members. Our results indicate that, though only relying on the constraints within each family (with four members) individually, PedPhase has better phasing accuracy than fastPHASE, even when the total numbers of genotyped individuals are the same. But for missing genotype imputation, fastPHASE performs better than PedPhase by taking population information into consideration. The relative influence of family constraints and population information on haplotyping accuracy as shown in this report provides some empirical bases on assessing the trade-off of genotyping family data under different settings.

## Background

To understand genetic variation among humans and to identify correlations between genetic variation and phenotypic variation (such as disease status, quantitative traits, etc.), it is necessary to understand haplotype structures in human populations [1]. However, the human genome is a diploid and, in practice, haplotype data are not collected directly, especially in large-scale sequencing projects, mainly due to cost considerations. Instead, genotype data are collected routinely in large sequencing projects. Hence, efficient and accurate computational methods and computer programs for the inference of haplotypes from genotypes are in high demand. The existing computational methods for haplotyping can be divided into two categories: statistical methods and rule-based (i.e., combinatorial) methods. Both approaches can be applied to pedigree or unrelated individual data. An earlier paper [2] has shown that the incorporation of pedigree data can improve haplotyping accuracy. But the conclusions are based on statistical criteria for a set of small number of markers (10–20), that is, how the haplotype frequencies in a short region are preserved. Here, we want to measure the accuracy of haplotype reconstruction for each individual for large number of markers (500).

We chose two representative programs for family and unrelated individual data and tested them on the Genetic Analysis Workshop 15 (GAW15) data set. PHASE [3] (and its latest version fastPHASE [4] is a popular statistical tool for large scale haplotype inference using population (of unrelated individuals) data. We have developed an efficient rule-based algorithm (PedPhase [5,6]) for haplotype inference from pedigree data that can output all optimal solutions with smallest number recombinants. The program HAPLORE [2,7] cannot handle data with recombinants, therefore it can not be used in this study. Furthermore, when the number of marker considered is in hundreds, it is highly likely that the haplotype solution in each family is unique. When the haplotype solution is unique in each family, results from PedPhase are optimal because it is an exact algorithm. The result of this report is based on the comparison of these two programs, fastPHASE V1.1 and PedPhase V2.1, using the simulated RA data from GAW15, in terms of accuracy, efficiency, and costs.

## Methods

### Data

Problem 3 contains 100 replicates of simulated RA data on 22 chromosomes. Each replicate includes 1500 nominally unrelated nuclear families of size 4 (2 parents and an affected sib pair (ASP)). To have significant linkage disequilibrium (LD) between markers, we have selected the single-nucleotide polymorphism (SNP) data of chromosome 6 from the first 10 replicates. The chromosome 6 data mimic a 300K-SNP array with 17,820 SNPs. We picked the first 500 loci from chromosome 6. From each replicate, the first 100, 200, and 400 individuals were selected, respectively, so as to form three trial samples. To examine the accuracy of methods for missing genotype inference, three mutated copies of each trial sample have been generated by randomly assigning a locus to be missing at probability 5%, 10%, and 15%. Finally, we had three variations of individual numbers (i.e., 100, 200, and 400), and four variations of missing rates (i.e., 0%, 5%, 10%, and 15%), a total of 12 testing categories. For each parameter combination, 10 independent replicates were selected, resulting in a total of 120 input trials.

### Comparison criteria

We compared the two methods in terms of accuracy, efficiency, and costs. Accuracy is measured by three criteria: the genotype inference error rate, the heterozygous switch error rate, and the point-wise error rate. We compared the reconstructed haplotype of each individual generated by these two programs against the actual haplotype or phase that is known from the original GAW15 data. The genotype inference error rate is the proportion of mistakenly inferred loci out of all missing loci. The heterozygous switch error rate is the proportion of mistakenly switched loci out of all heterozygous loci. Missing loci were ignored in computing the heterozygous switch error. The point-wise error rate is calculated allele-by-allele along each haplotype, yielding an overall score showing the difference of the generated haplotype against the original true haplotype taking every locus into account.

## Results

**Figure 1:**
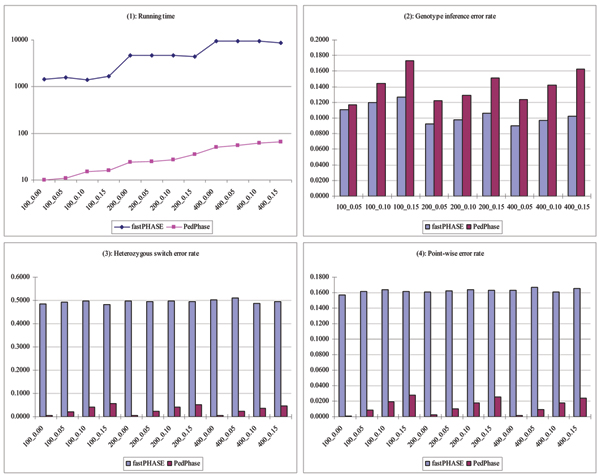
**Comparison of running time and error rates between fastPHASE and PedPhase on chromosome 6**. Panels 1, 2, 3, and 4 show the running time, genotype inference error rate, heterozygous switch error rate, and point-wise error rate of PedPhase and fastPHASE on different testing categories, e.g., 200_0.10 means 200 individuals with missing rate 0.1.

Results of fastPHASE and PedPhase on chromosome 6 are presented in Table 1 and Table 2, respectively. Detailed comparisons on each criterion are shown in Figure 1. As shown in Figure 1 (panel 1), PedPhase appears to be considerably faster than fastPHASE on the same number of individuals. PedPhase has a much lower heterozygous switch error rate and point-wise error rate, as shown in Figure 1 (panels 3 and 4). On average, they are approximately 10 times smaller than those from fastPHASE. Increasing sample sizes does not improve the accuracy for fastPHASE. However, better results in missing genotype imputation can be achieved for fastPHASE by incorporating information from population. For PedPhase, all three error rates increase as the missing rate increases. But those rates stay almost unchanged for different number of families. This is because PedPhase only exploits the constraints within a single family. The result of a specific family will be the same regardless of the existence of the other families. fastPHASE did not show any noticeable improvement over heterozygous switch error or point-wise error rate when we increased the number of individuals from 100 to 400. But its genotype inference accuracy improved slightly as the number of individuals increased. On the other hand, for a fixed sample size, fastPHASE only shows a gentle deterioration in genotype inference when we increased the missing rate from 5% to 15%.

**Table 1:** Running time and error rates of fastPHASE on chromosome 6

			Error rate		
			
Individuals	Missing	Running time (sec)	Genotype inference	Heterozygous switch	Point-wise
100	0.00	1437	------^a^	0.4835	0.1569
100	0.05	1563	0.1106	0.4926	0.1618
100	0.10	1380	0.1202	0.4967	0.1639
100	0.15	1671	0.1267	0.4829	0.1614
200	0.00	4679	------	0.4964	0.1609
200	0.05	4620	0.0922	0.4960	0.1620
200	0.10	4673	0.0979	0.4966	0.1635
200	0.15	4382	0.1064	0.4961	0.1631
400	0.00	9417	------	0.5035	0.1634
400	0.05	9411	0.0904	0.5093	0.1666
400	0.10	9360	0.0966	0.4880	0.1609
400	0.15	8524	0.1024	0.4959	0.1652

^a^------, Inference error rate is not measured because no missingness exists in this row.

**Table 2:** Running time and error rates of PedPhase on chromosome 6

			Error rate		
			
Individuals	Missing	Running time (sec)	Genotype inference	Heterozygous switch	Point-wise
100	0.00	10	------^a^	0.0061	0.0011
100	0.05	11	0.1165	0.0215	0.0088
100	0.10	15	0.1439	0.0412	0.0190
100	0.15	16	0.1731	0.560	0.0279
200	0.00	24	------	0.0058	0.0022
200	0.05	25	0.1223	0.0231	0.0099
200	0.10	27	0.1291	0.0398	0.0181
200	0.15	35	0.1513	0.0517	0.0256
400	0.00	51	------	0.0056	0.0015
400	0.05	55	0.1240	0.0232	0.0094
400	0.10	61	0.1418	0.0353	0.0179
400	0.15	66	0.1628	0.0459	0.0238

^a^------, Inference error rate is not measured because no missingness exists in this row.

## Discussion

The time complexity of fastPHASE is exponential to the input size as a result of the nature of its statistical approach. PedPhase, due to the use of integer linear programming, also has a time complexity exponential to the size of each family but it is linear to the number of nuclear families (it processes one family after another). Because the family size is fixed at 4 in this specific data set, PedPhase actually has a theoretical time complexity linear to the number of total families (input size). So it is not difficult to understand why fastPHASE is much slower than PedPhase.

As we examined into the output of fastPHASE, we noticed that a considerable proportion of its errors are block switch errors, which means that it actually generates quite a number of short but correct haplotype segments but it does not correctly fix the phase of these segments. That's why the heterozygous switch error rate and point-wise error rate of fastPHASE are much higher than those of PedPhase. This shows the importance of the use of family constraints in haplotype reconstruction. On the other hand, fastPHASE has better genotype inference performance than PedPhase. This robustness arises from its global consideration of all individuals such that there is much higher probability for missing data in one individual to be compensated by complete data in some other individuals. Therefore, for haplotype inference using pedigree data, it is necessary to take into consideration all the input families when imputing missing genotypes.

## Conclusion

The testing results have shown that even small families with size 4 can provide much information for haplotype reconstruction. Therefore, PedPhase performs better than fastPHASE in phasing accuracy (heterozygous switch error and point-wise error) on the given data set. The comparison of running time shows that methods working on small families do not necessarily cost more running time than methods on unrelated individuals while obtaining better phasing results. For genotype inference, the performance of fastPHASE is better than that of PedPhase, which shows that unrelated individuals contain most of the information that one should use in missing data imputation. The result also reflects the relative importance of family constraints and population information to haplotyping accuracy under different settings. Family constraints always help to increase the phasing accuracy, but may not be of significant help in missing data imputation if the missing rate is high. Because family information and population information are both important in haplotype inference, a method taking into account both types of information will be a necessary piece of future work in the development of haplotype inference algorithms.

## Competing interests

The author(s) declare that they have no competing interests.
